# Sentinel Case of *Candida auris* in the Western United States Following Prolonged Occult Colonization in a Returned Traveler from India

**DOI:** 10.1089/mdr.2018.0408

**Published:** 2019-05-30

**Authors:** Michael H. Woodworth, David Dynerman, Emily D. Crawford, Sarah B. Doernberg, Lynn Ramirez-Avila, Paula Hayakawa Serpa, Amy Nichols, Lucy M. Li, Amy Lyden, Cristina M. Tato, Steve Miller, Joseph L. Derisi, Charles Langelier

**Affiliations:** ^1^ Division of Infectious Diseases, Department of Medicine, Emory University School of Medicine, Atlanta, Georgia.; ^2^ Chan Zuckerberg Biohub, San Francisco, California.; ^3^ Division of Infectious Diseases, Department of Medicine, University of California, San Francisco, San Francisco, California.; ^4^ Division of Pediatric Infectious Diseases and Global Health, Department of Pediatrics, University of California, San Francisco, San Francisco, California.; ^5^ Hospital Epidemiology and Infection Control, University of California, San Francisco, San Francisco, California.; ^6^ Department of Laboratory Medicine, University of California, San Francisco, San Francisco, California.; ^7^ Department of Biochemistry and Biophysics, University of California, San Francisco, San Francisco, California.

**Keywords:** *Candida auris*, emerging infection, antimicrobial resistance, echinocandin resistance, metagenomic

## Abstract

*Candida auris* is an emerging multidrug-resistant yeast with high mortality. We report the sentinel *C. auris* case on the United States West Coast in a patient who relocated from India. We identified close phylogenetic relatedness to the South Asia clade and *ERG11* Y132F and *FKS1* S639Y mutations potentially explaining antifungal resistance.

## Introduction

*C**andida auris* is an emerging fungal pathogen with high minimum inhibitory concentrations (MICs) for many antifungals. Since identification in 2009, it is increasingly recognized as an important cause of invasive disease and nosocomial outbreaks, with high-associated in-hospital mortality of 40–72%.^1^ Genomic evaluation of strains from multiple geographic regions suggests simultaneous emergence of distinct geographic clades on three continents, as opposed to dissemination from a single source.^1^ This observation suggests that environmental factors such as increased antifungal use may have contributed to *C. auris* emergence.^1^

In addition to high fluconazole MICs, *C. auris* isolates also frequently have high MICs for other antifungals, including amphotericin and less frequently echinocandins.^1^ Despite the alarming frequency of elevated antifungal MICs in *C. auris*, the underlying mechanisms and alleles associated with this resistance have not been fully characterized. In *C. auris* as well as in other *Candida* species, mutations in *ERG11* (ergosterol synthetase), *FKS1* (1,3 beta-d-glucan synthetase), and *FUR1* (uracil phosphoribosyltransferase) have been associated with resistance to fluconazole, echinocandins, and flucytosine, respectively.^1,2^ Previous studies suggest that mutations in these genes can arise in the setting of systemic antifungal therapy.^3^

Despite first appearing in the eastern United States in 2013, *C. auris* had not been detected on the United States West Coast.^2^ In this study, we report the identification of *C. auris* in this region, which was unusual in that it did not establish endemicity, and use whole-genome sequencing (WGS) to identify strain origin and evaluate genetic mechanisms of antifungal resistance.

## Methods

### Case description

An elderly man with metastatic rectal cancer relocated from India to California. He had received chemotherapy and radiation while in India and had so undergone intraabdominal surgeries complicated by sepsis. In the year following his move, he required multiple admissions to the University of California, San Francisco (UCSF) Medical Center for management of his malignancy and for secondary infections with carbapenem-resistant Enterobacteriaceae (CRE), for which he was placed in contact isolation. During his initial multi-month admission, two cultures from his urostomy grew 10,000 colony-forming units of a non-*Candida albicans* yeast that was not further speciated due to unclear clinical significance. In the course of his care, he was treated with echinocandins with prophylactic intent. Several months after initial admission, he was transitioned to palliative care. Three days before death, a nephrostomy culture returned positive for yeast, which was ultimately speciated as *C. auris.*

### Clinical microbiology and antifungal susceptibility testing

Urine collected from the patient's nephrostomy tube into a sterile container underwent quantitative culture for bacteria and yeast using standard culture methods. Species identification was made using matrix-assisted laser desorption ionization-time of flight mass spectrometry (Brucker Diagnostics), which returned a score value of 2.14, and was additionally confirmed by the California Department of Public Health. Antifungal susceptibility testing was performed using Sensititre YeastOne MIC plates (Trek Diagnostic Systems, Inc.), which has >95% agreement with the Clinical Laboratory Standards Institute reference method.^4^

### Whole-genome sequencing

DNA was extracted from the cultured *C. auris* isolate using the Zymo ZR Bacterial/Fungal DNA kit. Library preparation was completed with the New England Biolabs NEBNext Ultra II DNA library prep kit and WGS was performed using an Illumina NextSeq. The same DNA also underwent library prep using the Oxford Nanopore Rapid Low Input by PCR Barcoding Kit and WGS on a MinION instrument.

### Genome assembly, phylogenetic analyses, and antifungal resistance gene analysis

Raw Illumina sequencing reads were quality filtered using PriceSeqFilter^5^ and then parsed with Nanopore reads for hybrid *de novo* assembly using DBG2OLC.^6^ Reference-based whole-genome phylogenetic analysis constructed from core genome single-nucleotide polymorphisms (SNPs) was carried out with the Northern Arizona SNP Pipeline^7^ using Pakistan strain B8441 as the reference genome and incorporating genomes from Lockhart *et al.*^1^ as well as *C. auris* isolate 16B15b containing the *FKS1* S639P mutation identified by Rhodes *et al.*^3^ RAxML-ng^8^ was used to build maximum likelihood phylogenetic trees as detailed in Supplementary Methods (see Supplementary Tables S2–S4). To identify genetic mutations associated with fluconazole or echinocandin resistance, Illumina sequences were aligned against *ERG11* (GenBank KY410388.1) and *FKS1* (GenBank XM_018312471.1) using BowTie2.^9^ Mutations were confirmed by *ERG11* and *FKS1* PCR followed by Sanger Sequencing (Supplementary Table S1) following previously described methods.^10^

## Results

### Assembly and phylogenetic characteristics

**FIG. 1.**
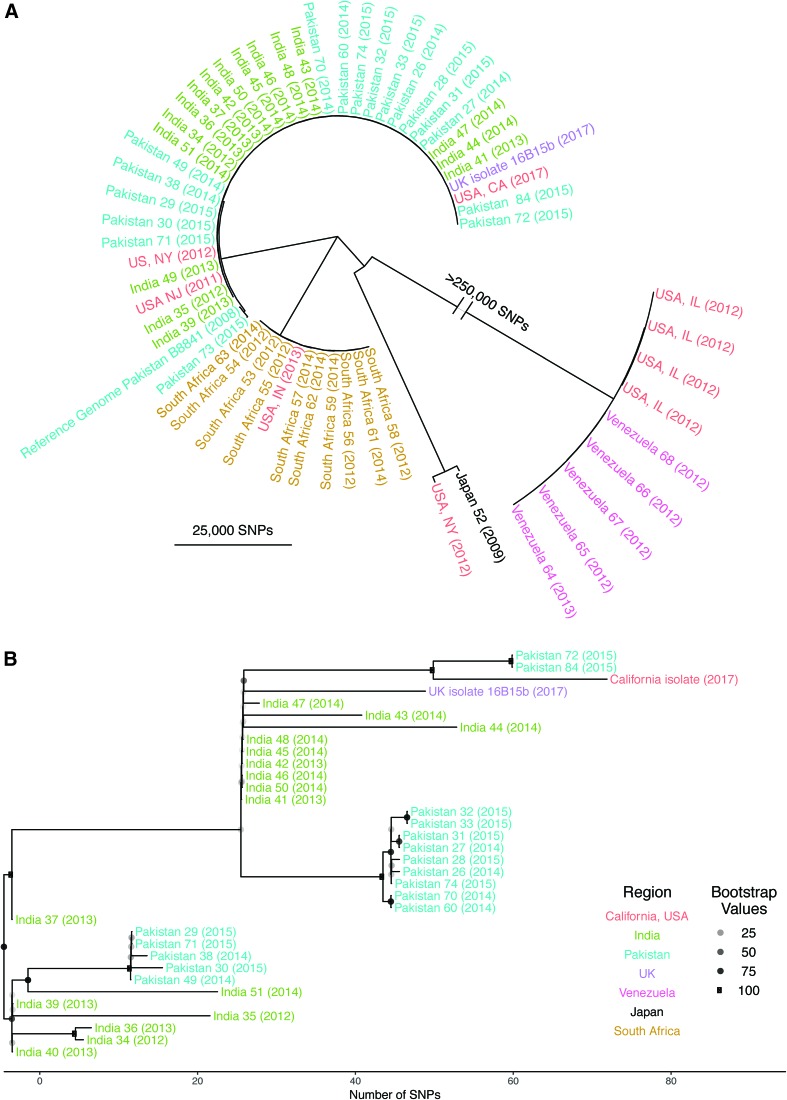
**(A)** Phylogenetic assessment based on core genome single-nucleotide polymorphisms demonstrated the four known geographic clades^1^ and placed the California isolate within the South Asia clade. **(B)** Detailed phylogenetic tree describing the South Asia clade, including the California isolate and United Kingdom outbreak isolate16B15b,^3^ which both harbored the *FKS1* S639P mutation. Color images are available online.

*De novo* hybrid assembly of Illumina and Oxford Nanopore reads produced a total of 33 contigs spanning 12 Mb, characterized by 44.9% GC content, consistent with prior estimates.^1,3^ Whole-genome phylogenetic analysis based on a core genome of 208,384 SNPs placed this isolate within the South Asia clade (Fig. 1A). On average, 56 SNPs separated this isolate from others from the South Asia clade (Fig. 1B).

### Phenotypic and genotypic assessment of antifungal resistance

The California isolate demonstrated low MICs to amphotericin (1 μg/mL), flucytosine (0.5 μg/mL), and voriconazole (0.032 μg/mL). The isolate had an elevated fluconazole MIC of 32 μg/mL. Assessment of this isolate's *ERG11* (encoding ergosterol synthetase) allele revealed the well-characterized Y132F substitution in the azole resistance hotspot region.^1,3^ Unlike most *C. auris* strains, this California isolate also exhibited a high caspofungin MIC of 8 μg/mL. Interrogation of *FKS1* [encoding (1,3)-β-d-glucan synthetase] revealed a S639Y mutation in the echinocandin resistance hotspot 1 region (Supplementary Table S1).^3,10^

## Discussion

### *C. auris* emerges on the West Coast of the United States

In this study, we report the first case of *C. auris* on the United States West Coast, a region that had no previous reports of the pathogen despite emergence in New York in 2013. The patient's history of health care exposure in India combined with the clustering of his *C. auris* isolate with the South Asia clade by WGS phylogenetic analysis suggests that he acquired *C. auris* abroad before hospitalization in California. This finding supports current guidance from the United States Centers for Disease Control and Prevention to speciate all *Candida* in high-risk patients, including those from regions of high *C. auris* prevalence, to allow for early implementation of infection control measures.^1,11^ Following identification of *C. auris*, enhanced infection control measures were implemented at UCSF, including surface disinfection, a unit-level point prevalence survey, and prospective surveillance. No additional cases of *C. auris* at our medical center have been identified in over a year. This case represents an unusual interruption in spread and prolonged health care environmental contamination that has been characteristic of detection of health care-associated *C. auris.* Early implementation of contact precautions for CRE may have contributed to curbing transmission of *C. auris* in this case.

This isolate had a high fluconazole MIC with an observed *ERG11* Y132F mutation.^1,3^ The California *C. auris* isolate also demonstrated a high echinocandin MIC, which is observed in <10% of *C. auris* strains.^1^ It is possible that this patient's prophylactic treatment with echinocandins could have selected for resistance as observed in this isolate. This *C. auris* isolate also had a *FKS1* hotspot-1 region mutation, which has been associated with echinocandin resistance in multiple other *Candida* species.^3,10^ The identified *FKS1* S639 substitutions of nonpolar residues (Y, F, P) has also been identified in other *C. auris* strains with high echinocandin MIC values, suggesting a key role for this amino acid in echinocandin resistance.^3,10^

Further study is needed to estimate the prevalence and duration of colonization by this emerging pathogen. Future work using WGS is needed to clarify the origins of *C. auris*, transmission patterns, and mechanisms of resistance to prevent and manage this emerging fungal pathogen of global significance.

## Data Availability

Raw sequences are available via BioProject ID PRJNA480539.

## Supplementary Material

Supplemental data
