# Supplementary material for: Sentinel Case of Candida auris in the Western United States Following Prolonged Occult Colonization in a Returned Traveler from India
Source: Microb Drug Resist. 2019 May 30;25(5):677–80. doi: 10.1089/mdr.2018.0408 (PMC6555181; doi:10.1089/mdr.2018.0408)
Supplement: Supplemental data [file Supp_Data.pdf]

Supplementary Data

Supplementary Methods

Phylogenetic analysis was carried out by evaluating single-nucleotide polymorphisms (SNPs) between isolates using the Northern Arizona SNP Pipeline (NASP) pipeline version 1.0.0.[S1] In NASP, we used *Candida auris* strain B8841 assembly GCA 002759435.2 as the reference genome. We used BWA MEM 0.7.5a-r405[S2] to align reads from the Lockhart *et al.* study[S3] (Supplementary Table S2), this California isolate (BioProject ID PRJNA480539), additional isolates from the United States (Supplementary Table S3), the United Kingdom outbreak isolate 16B15b (Accession ERR2299890) containing the *FKS1* S639P mutation identified by Rhodes *et al.*,[S4] and six available draft genome assemblies (Supplementary Table S4). We used Samtools[S5] version 0.1.19-44428cd to call SNPs from the alignments.

NASP produced a multi-FASTA alignment containing 210,456 SNPs present across the samples. We ran jModelTest2[S6] which identified the transversion model (TVM) as the best fitting nucleotide substitution model for our alignment according to the Akaike information criterion. We then ran RAxML-ng v0.6.0 BETA[S7] in ''all-in-one'' mode which produced a phylogenetic tree using a maximum likelihood tree search followed by a nonparametric bootstrap with 100 replicates. We specified the TVM model and the ''—all'' option to enable ''all-in-one'' mode. All other RAxML options were left at their defaults. RAxML was used to build a maximum likelihood phylogenetic trees,[S8] which were visualized with Dendroscope,[S9] FigTree,[S10] and Geneious[S11] software packages. The maximum likelihood tree was visualized using the R[S12] (www.R-project.org) package ggtree[S13] v3.8 (DOI: 10.1093/molbev/msy194).

To produce the detailed phylogenetic tree in Fig. 1B, we re-ran the above NASP pipeline on a subset of the above dataset consisting of reads from India/Pakistan, this California isolate, and United Kingdom isolate 16B15b. We also included the South Africa 55 (2012) sample (Supplementary Table S2) as an outgroup to root the resulting tree. In this study, the NASP pipeline produced a multi-FASTA alignment containing 45,573 SNPs. We ran the remaining steps of the analysis identically as above. The phylogeny in Fig. 1B did not include Pakistan Reference Genome B8841 (2008) and Pakistan 73 (2015) isolates that were more distantly related to the rest of the India/Pakistan clade.

Supplementary Table S1. Nucleotide Sequences Surrounding the ERG11 Y132D and FKS1 S639Y Hotspot Region Mutations in the California *Candida auris* Isolate Determined by Illumina Sequencing, Nanopore Sequencing, and Confirmed by Sanger Sequencing of PCR-Amplified Genes

ERG11 **Y132F**
AAA GGT GTC ATT TTC GAC TGT CCC AAC
K G V I F D C P N
FKS1 **S639Y**
TTC TTG ACT TTG TAC TTG AGA GAT CCT
F L T L Y L R D P

Light gray shading, alternating for readability, indicates nucleotides encoding each translated amino acid *below*, with mutations highlighted in *black*.

Source: Chowdhary *et al.*, 2018.[S14]

Supplementary Table S2. Sequencing Reads from Lockhart *et al.* *Candida auris* Isolates Used in Phylogenetic Analyses

SRA Run ID	*Location (Date)*
SRR3883426	Pakistan 26 (2014)
SRR3883427	Pakistan 27 (2014)
SRR3883428	Pakistan 28 (2015)
SRR3883429	Pakistan 29 (2015)
SRR3883430	Pakistan 30 (2015)
SRR3883431	Pakistan 31 (2015)
SRR3883432	Pakistan 32 (2015)
SRR3883433	Pakistan 33 (2015)
SRR3883434	India 34 (2012)
SRR3883435	India 35 (2012)
SRR3883436	India 36 (2013)
SRR3883437	India 37 (2013)
SRR3883438	Pakistan 38 (2014)
SRR3883439	India 39 (2013)
SRR3883440	India 40 (2013)
SRR3883441	India 41 (2013)
SRR3883442	India 42 (2013)
SRR3883443	India 43 (2014)
SRR3883444	India 44 (2014)
SRR3883445	India 45 (2014)
SRR3883446	India 46 (2014)
SRR3883447	India 47 (2014)
SRR3883448	India 48 (2014)
SRR3883449	Pakistan 49 (2014)
SRR3883450	India 50 (2014)
SRR3883451	India 51 (2014)
SRR3883452	Japan 52 (2009)
SRR3883453	South Africa 53 (2012)
SRR3883454	South Africa 54 (2012)
SRR3883455	South Africa 55 (2012)
SRR3883456	South Africa 56 (2012)
SRR3883457	South Africa 57 (2014)
SRR3883458	South Africa 58 (2014)
SRR3883459	South Africa 59 (2014)
SRR3883460	Pakistan 60 (2014)
SRR3883461	South Africa 61 (2014)
SRR3883462	South Africa 62 (2014)
SRR3883463	South Africa 63 (2014)
SRR3883464	Venezuela 64 (2013)
SRR3883465	Venezuela 65 (2012)
SRR3883466	Venezuela 66 (2012)
SRR3883467	Venezuela 67 (2012)
SRR3883468	Venezuela 68 (2012)
SRR3883470	Pakistan 70 (2014)
SRR3883471	Pakistan 71 (2015)
SRR3883472	Pakistan 72 (2015)
SRR3883473	Pakistan 73 (2015)
SRR3883474	Pakistan 74 (2015)
SRR6220384	Pakistan 84 (2015)

Source: Lockhart *et al.*, 2017.[S3]

Supplementary Table S3. Sequencing Reads from Additional *Candida auris* Isolates from the United States Used in Phylogenetic Analyses

SRR7909157	United States of America, NY (2012)
SRR7909137	United States of America, NJ (2011)
SRR7909359	United States of America, IN (2013)
SRR7909305	United States of America, NY (2012)
SRR7909220	United States of America, IL (2012)
SRR7909151	United States of America, IL (2012)
SRR7909238	United States of America, IL (2012)
SRR7909335	United States of America, IL (2012)

Source: Chow *et al*., 2018.[S15]

Supplementary Table S4. Draft Genome Assemblies Used in Phylogenetic Analyses

Strain	*Assembly*	*Scaffolds*
6684	GCA_001189475.1	99
B8441	GCA_002759435.2	15
B11221	GCA_002775015.1	20
B11220	GCA_003013715.1	320
B11243	GCA_003014415.1	238
	GCA_001049995.1	533

Supplementary References

S1. Roe, C., D.E. Smith, C.H.D. Williamson, M. Aziz, P. Keim, C.M. Hepp, E.M. Driebe, D. Lemmer, J. Travis, N.D. Hicks, J.M. Schupp, D.M. Wagner, D.M. Engelthaler, J.D. Gillece, J.W. Sahl, and K.P. Drees. 2016. NASP: an accurate, rapid method for the identification of SNPs in WGS datasets that supports flexible input and output formats. Microb. Genomics 2:e000074.

S2. Li, H., and R. Durbin. 2009. Fast and accurate short read alignment with Burrows-Wheeler transform. Bioinformatics 25:1754–1760.

S3. Lockhart, S.R., K.A. Etienne, S. Vallabhaneni, J. Farooqi, A. Chowdhary, N.P. Govender, A.L. Colombo, B. Calvo, C.A. Cuomo, C.A. Desjardins, E.L. Berkow, M. Castanheira, R.E. Magobo, K. Jabeen, R.J. Asghar, J.F. Meis, B. Jackson, T. Chiller, and A.P. Litvintseva. 2017. Simultaneous emergence of multidrug-resistant *Candida auris* on 3 continents confirmed by whole-genome sequencing and epidemiological analyses. Clin. Infect. Dis. 64:134–140.

S4. Rhodes, J., A. Abdolrasouli, R.A. Farrer, C.A. Cuomo, D.M. Aanensen, D. Armstrong-James, M.C. Fisher, and S. Schelenz. 2018. Genomic epidemiology of the UK outbreak of the emerging human fungal pathogen *Candida auris*. Emerg. Microbes Infect. 7:43.

S5. Li, H., B. Handsaker, A. Wysoker, T. Fennell, J. Ruan, and N. Homer. 2009. The sequence alignment/map format and SAMtools. Bioinformatics 25:2078–2079.

S6. Darriba, D., G.L. Taboada, R. Doallo, and D. Posada. 2012. jModelTest 2: more models, new heuristics and parallel computing. Nat. Methods 9:772–772.

S7. Kozlov, A. amkozlov/raxml-ng: RAxML-NG v0.6.0 BETA (Version 0.6.0). Zenodo. http://doi.org/10.5281/zenodo.1291478 (accessed June 16, 2018).

S8. Stamatakis, A. 2014. RAxML version 8: a tool for phylogenetic analysis and post-analysis of large phylogenies. Bioinformatics 30:1312–1313.

S9. Huson, D.H., and C. Scornavacca. 2012. Dendroscope 3: an interactive tool for rooted phylogenetic trees and networks. Syst. Biol. 61:1061–1067.

S10. Rambaut, A. (2012). FigTree. Univ Oxf Oxf UK [Internet]. (v1.4.0). Available at http://tree.bio.ed.ac.uk/software/figtree (accessed July 11, 2018).

S11. Kearse, M., R. Moir, A. Wilson, S. Stones-Havas, M. Cheung, and S. Sturrock. 2012. Geneious Basic: an integrated and extendable desktop software platform for the organization and analysis of sequence data. Bioinformatics 28:1647–1649.

S12. R Core Team. 2018. R: A language and environment for statistical computing. R Foundation for Statistical Computing, Vienna, Austria. http://www.R-project.org/

S13. Yu, G., T.T.-Y. Lam, H. Zhu, and Y. Guan. 2018. Two Methods for Mapping and Visualizing Associated Data on Phylogeny Using *Ggtree*. Mol. Biol. Evol. 35:3041–3043.

S14. Chowdhary, A., A. Prakash, C. Sharma, M. Kordalewska, A. Kumar, S. Sarma, B. Tarai, A. Singh, G. Upadhyaya, S. Upadhyay, P. Yadav, P.K. Singh, V. Khillan, N. Sachdeva, D.S. Perlin, and J.F. Meis. 2018. A multicentre study of antifungal susceptibility patterns among 350 *Candida auris* isolates (2009–17) in India: role of the ERG11 and FKS1 genes in azole and echinocandin resistance. J. Antimicrob. Chemother. 73:891–899.

S15. Chow, N.A., L. Gade, S.V. Tsay, K. Forsberg, J.A. Greenko, K.L. Southwick, P.M. Barrett, J.L. Kerins, S.R. Lockhart, T.M. Chiller, A.P. Litvintseva AP, and US *Candida auris* Investigation Team. 2018. Multiple introductions and subsequent transmission of multidrug-resistant *Candida auris* in the USA: a molecular epidemiological survey. Lancet Infect. Dis. 18:1377–1384.
